# Flavonoid Composition and Biological Activities of Ethanol Extracts of* Caryocar coriaceum* Wittm., a Native Plant from Caatinga Biome

**DOI:** 10.1155/2017/6834218

**Published:** 2017-09-07

**Authors:** Daniela Ribeiro Alves, Selene Maia de Morais, Fernanda Tomiotto-Pellissier, Milena Menegazzo Miranda-Sapla, Fábio Roger Vasconcelos, Isaac Neto Goes da Silva, Halisson Araujo de Sousa, João Paulo Assolini, Ivete Conchon-Costa, Wander Rogério Pavanelli, Francisco das Chagas Oliveira Freire

**Affiliations:** ^1^Veterinarian Sciences Post Graduation Program, Ceará State University, Av. Dr. Silas Munguba 1700, Campus do Itaperi, 60714-903 Fortaleza, CE, Brazil; ^2^Pathological Sciences Center, Londrina State University, Rodovia Celso Garcia Cid, PR 445, Km 380, Campus Universitário, 86057-970 Londrina, PR, Brazil; ^3^Embrapa Agroindústria Tropical, Rua Dra. Sara Mesquita 2270, Planalto do Pici, 60511-110 Fortaleza, CE, Brazil; ^4^Chemical Course, Ceará State University, Av. Dr. Silas Munguba 1700, Campus do Itaperi, 60714-903 Fortaleza, CE, Brazil

## Abstract

*Caryocar coriaceum* fruits, found in Brazilian Cerrado and Caatinga, are commonly used as food and in folk medicine, as anti-inflammatory, bactericide, fungicide, leishmanicide, and nematicide. Due to the biological potential of this plant, this study focuses on the evaluation of antifungal and antileishmanial activities, including anticholinesterase and antioxidant tests, correlating with total phenols and flavonoids content. Peel extracts contain higher yield of phenols and flavonoids as analyzed by spectrophotometric methods. HPLC analysis of flavonoids revealed that isoquercitrin is the main flavonoid in both parts of the fruit, and peel extract showed the best antioxidant activity. In the inhibition of the acetylcholinesterase assay, both extracts demonstrate action comparable to physostigmine. The antimicrobial activity of extracts was evaluated against strains of* Malassezia* sp. and* Microsporum canis*, using the broth microdilution technique, in which the extracts showed similar MIC and MFC. The extracts present antileishmanial activity and low toxicity on murine macrophages and erythrocytes. Therefore, these results suggest a potential for the application of* C. coriaceum* fruit's ethanol extracts in the treatment against dermatophyte fungi and leishmaniasis, probably due to the presence of active flavonoids. Further in vivo studies are recommended aiming at the development of possible new pharmaceutical compounds.

## 1. Introduction

In veterinary care, some diseases are common in Brazil as fungal dermatophytosis and cutaneous/mucocutaneous leishmaniasis. Regarding fungal diseases,* Malassezia, Microsporum*, and* Trichophyton* genera are the main infectious agents of human and animal cutaneous mycoses [1, 2].

Leishmaniases are zoonosis caused by protozoa of the* Leishmania* genus with a wide range of clinical symptoms: cutaneous, mucocutaneous, and visceral [3]. In these parasitic diseases, humans and wild, synanthropic, and domestic mammals act as hosts and/or reservoirs of several* Leishmania* spp. [4].

Cutaneous/mucocutaneous leishmaniasis is usually characterized by chronic skin lesions and permanent scars with deformation of the infected area [5]. This disease presented more than 1 million cases reported in the last five years, with over 431 million people living in endemic areas at risk of infection [3].* Leishmania* spp. are pathogens that infect mainly macrophages, but also neutrophils and dendritic cells. The parasites are able to evade the microbicidal mechanism of these cells resulting in the different forms of disease, according to the* Leishmania* species [6–8].

Although both diseases present therapeutic options such as the azoderivatives for fungal treatment, especially ketoconazole and itraconazole and pentavalent antimonial drugs for leishmaniasis chemotherapy, these drugs present low efficacy and severe side effects as cardiotoxicity and hepatotoxicity [5]. Thus, many researchers have been trying to find safer plant-derived natural products to treat these diseases [6, 7, 9, 10].

The* Caryocar* genus, known popularly as Pequi, has a wide distribution and is represented in several Brazilian biomes such as Cerrado, the Atlantic Forest, Amazon, and Caatinga [11, 12]. The fruit pulp is largely used in food preparation, mainly with rice. The fruit pulp and seed oils of* Caryocar coriaceum* have been used in wound healing, as an anti-inflammatory agent, and for the treatment of diseases of the respiratory tract, including cough, bronchial affections, and asthma The fruit crude oil is also used on small skin wounds, in the form of compresses and massage, for treatment of rheumatic and muscular pains [13].

The* Caryocar* species is considered a promising medicinal product due to its bactericidal, fungicidal, leishmanicidal, and nematicidal activities [14, 15]. In this work, ethanol extracts of pulp and peel of* C. coriaceum* fruits were evaluated in relation to phenols and flavonoids content and biological activities such as antifungal and antileishmanial activities using different microorganisms from the previous work. Thus, in this study, dermatophyte fungi and* Leishmania amazonensis* parasites were used and the antioxidant and antiacetylcholinesterase activities were investigated, which support their antileishmanial and antifungal properties.

## 2. Materials and Methods

### 2.1. Chemicals

2,2-Diphenyl-1-picrylhydrazyl (DPPH), 5,5′-dithiobis(2-nitrobenzoic acid) (DTNB), acetylthiocholine iodide (ATCI), and other reagents were acquired from Sigma Chemical Co. (St. Louis, MO, USA). Quercetin, isoquercitrin, and rutin were obtained from the seeds of the plant* Dimorphandra gardneriana* according to a previous report [16].

### 2.2. Preparation of Plant Extracts

Peel and pulp of* C. coriaceum* mature fruits were obtained at the Campus of the Ceará State University (lat.: −3.792222; long.: −38.556111), Fortaleza, Brazil. These* C. coriaceum* plants were submitted and identified by Prisco Bezerra Herbarium under the code EAC57060. The extracts were obtained by cold maceration with 96% ethanol, at 12 h cycle of light, without agitation for 7 days. Filtration of the supernatant and evaporation of the solvent at reduced pressure in a rotary evaporator led to crude ethanol extracts of Pequi fruit pulp and fruit peel.

### 2.3. Qualitative Determination of Chemical Constituents

The presence of secondary metabolites was detected by visual observation of color changes or precipitate formation reactions [17]. The reactions were conducted by the pH variation of ethanol extracts (with sodium hydroxide and sulfuric acid) to detect the presence of flavonols, flavanones, flavanonols, anthocyanins, and catechins; Lieberman-Burchard reagent (acetic anhydride plus sulfuric acid) is used for steroids (green color) or triterpenes (red color) characterization. Ferric chloride solution was used to detect phenols and tannins, and Shinoda reagent (concentrated HCl and granulated magnesium ribbon) was used to detect flavonoids and xanthones (a pink to red color); when shaking the dry extract with distilled water, if permanent foam is formed, this characterizes the presence of saponins.

### 2.4. Quantitative Determination of Total Phenol Content

Total phenol content was quantitatively determined using Folin–Ciocalteu's method [18]. The absorbance was measured at 750 nm using a UV/Vis spectrophotometer. The blue color indicated the presence of phenol content. The results are expressed in mg of gallic acid equivalent per gram of extract (mg GAE/g) based on a linear equation for a standard curve prepared with gallic acid.

### 2.5. Quantitative Determination of Total Flavonoid Content

The flavonoid content was determined using Funari and Ferro's method [19]. The absorbance was measured at 425 nm. Yellow color indicated the presence of flavonoids. The flavonoid content is expressed in mg of quercetin equivalent per gram of extract (mg EQ/g), on a linear equation for a standard curve prepared with quercetin.

### 2.6. Characterization of Flavonoids by HPLC

The identification and quantification of flavonoids on EEPUCC and EEPECC were performed by high-performance liquid chromatography (HPLC) with Shimadzu liquid chromatograph coupled to an SCL-10AVP controller system, UV-Vis detector SPD-10AVP, and isocratic pump LC-10ATVP. The LC Solution software was used to record the chromatograms and measure peak areas. The column used was a Shimadzu analytical CLC-ODS M (C-18) of 25 cm. The calibration curve was constructed using the standards rutin, isoquercitrin, and quercetin, injected at different concentrations (0.25, 0.05, 0.025, and 0.005 mg/mL) into the liquid chromatograph. The flow rate was 1.8 mL per minute for quercetin and 1.25 mL per minute for rutin at a wavelength of 350 nm a mobile phase composed of acetonitrile and H_3_PO_4_ buffer at pH 2.8 (20% : 80%). The linear regression equation was obtained by using the Microsoft Office Excel 2010 program. The chromatographic profiles of flavonoids rutin, quercetin, and isoquercitrin, the chosen standards, were obtained by preparing ethanol solutions at a concentration of 0.5 mg/mL and then injecting them into the high-performance liquid chromatograph. As the mobile phase, the same solution was used for calibration curve at the same wavelength and flow rate of 1.80 mL per minute.

### 2.7. Assessment of Antioxidant Activity via 2,2-Diphenyl-1-picrylhydrazyl (DPPH) Radical Reduction

Antioxidant activity of EEPUCC and EEPECC was assessed using a previously described method [20], with some modifications. Several dilutions of the samples and positive control (rutin) in methanol were prepared to obtain the concentrations of 100, 50, 5, and 1 *µ*g/mL. Methanol was used as a negative control. The absorbances were measured at 515 nm using a UV-Vis spectrophotometer. The percentage inhibition (PI) was calculated according to the following equation: PI% = [(absorbance of DPPH − absorbance of the extract)/absorbance of DPPH] × 100. The IC_50_ values were determined by linear regression of the plotted data followed by Tukey's test for multiple comparisons.

### 2.8. Inhibition of Acetylcholinesterase (AChE) Enzyme

The AChE inhibitory activity was qualitatively assessed using Ellman's [21] methodology, adapted for thin layer chromatography by Rhee et al. [22]. Solutions of the fruit pulp and peel at the concentration of 2.0 mg/mL were applied to TLC aluminum chromatoplate silica gel 60 F254 (Merck®) forming 2 mm spots. The plate was sprayed with 5,5′-dithiobis(2-nitrobenzoic acid) (DTNB)/acetylthiocholine iodide (ATCI) reagent (1 mM DTNB and 1 mM ATCI in 50 mM Tris-HCl, pH 8) until the silica was carefully saturated with the solvent. Plates were allowed to dry and then 5 U/mL of AChE enzyme solution was sprayed. A yellow background was observed, with white zones, indicating the presence of AChE enzyme inhibiting compounds. These zones became visible after 5 min. The zones were observed, measured, and recorded. Physostigmine was used as standard.

### 2.9. Fungicidal Assay

The minimum concentration capable of inhibiting 100% fungi growth (MIC) was determined by the dilution technique, according to CLSI method [23]. Six strains were tested (3* Malassezia* spp.: MA239, MA276, and MA355; 3* Microsporum canis*: MC017/15, MC029/15, and MC115/15), isolated from infected domestic animals, identified and stocked at the Microbiology Section, and kindly donated by Vettings®. The spore suspension solution for initial inoculation was prepared from filamentous fungi cultivated on potato dextrose agar (PDA) and incubated at a temperature of 28 ± 2°C for 7 days. The spore count was performed in a Neubauer chamber to achieve the concentration of 10^5^ to 10^6^ cells. In laminar flow cabinet, 100.0 *μ*L of the RPMI medium was distributed into each well of a 96-well microplate. 100.0 *μ*L of EEPUCC and EEPECC extracts was added and serial dilution was performed from 2500.0 to 2.44 *μ*g/mL. Finally, 50.0 *μ*L of the fungal suspension was added to all wells except the lines intended for the control of the sterile medium. The readings were taken by checking the MIC, with the aid of stereoscopically checking the lowest concentration of the samples capable of inhibiting 100% of the growth of the microorganism, after 5 days of incubation. The plates were also inspected under an inverted microscope to ensure growth of the controls and sterile conditions. The minimal fungicide concentration (MFC), considered as the minimum concentration capable of killing 100% of fungi, was measured by transferring 50.0 *μ*L from wells without fungal growth and inoculating on PDA. MFC was established according to the fungus growth after incubation under the same conditions for 5 days.

### 2.10. *Leishmania* Parasite

*Leishmania (Leishmania) amazonensis* (MHOM/BR/1989/166MJO) was used in promastigote forms, in the stationary growth phase (day 5 of culture). The parasites were obtained from popliteal lymph nodes of* L. amazonensis*-infected BALB/c mice and maintained in 199 culture medium (Gibco) supplemented with 10% fetal bovine serum (FBS) (Gibco), 10 mM HEPES Biological Buffer (AMRESCO), 0.1% human urine, 0.1% L-glutamine (SYNTH), penicillin (10 U/mL) and streptomycin (10 *μ*g/mL) (Gibco), and 10% sodium bicarbonate (SYNTH). Cell cultures were incubated at 25°C in 25 cm^2^ flasks. All parasites were from a culture that was serially passed for less than 5 weeks.

### 2.11. Viability of* L. amazonensis* Promastigote Forms

The direct effect of EEPUCC and EEPECC extracts against* L. amazonensis* was assessed in 24-well microtiter plates, each well containing 1000 *μ*L of 199 supplemented culture medium with 1 × 10^6^ promastigote forms in stationary phase with or without the extracts of interest at final concentrations of 0.1, 0.05, and 0.025 mg/mL. Viable promastigote concentration was determined by Neubauer chamber counting after 24, 48, and 72 h of treatment. In the stock solutions of extracts, 0.01% dimethyl sulfoxide (DMSO) (Gibco) was used as vehicle. Untreated parasites and vehicle only (0.01% DMSO) were included as negative controls. The plates were also inspected under an inverted microscope to ensure growth of the controls and sterile conditions. The 50% inhibitory concentration (IC_50_) was determined by nonlinear regression analysis of the obtained data.

### 2.12. Animals

BALB/c mice weighing approximately 25–30 g and aged 6–8 weeks were used, according to protocols approved by the ethics committee of Londrina State University, which approved the protocol for animal use (13134.2016.62).

### 2.13. Peritoneal Macrophages Viability Assay

The viability of peritoneal macrophages treated with EEPUCC and EEPECC extracts was evaluated using the 3-(4,5-dimethylthiazol-2-yl)-2,5-diphenyltetrazolium bromide (MTT) assay as previously described by Mosmann [24]. BALB/c peritoneal macrophages (5 × 10^5^ U/mL) were cultured in 24-well plates with 500 *μ*L of 199 medium for 2 h for adherence at 37°C and 5% CO_2_. The cells were washed with PBS and then adherent cells were incubated with different concentrations of extracts (2.5–0.025 mg/mL) or with vehicle (0.1% DMSO) and maintained in culture for 24 h at 37°C and 5% CO_2_. After incubation with extracts, the macrophages were washed with PBS and MTT was added at a final concentration of 5 *μ*g/mL in each well, followed by incubation for 4 h at 37°C/5% CO_2_. The MTT formazan product was solubilized with 300 *μ*L of DMSO, and plates were read at 570 nm in a spectrophotometer. The 50% cytotoxicity concentration (CC_50_) was determined by nonlinear regression analysis of the obtained data.

### 2.14. Selectivity Index (SI)

The degree of selectivity of EEPUCC and EEPECC extracts was expressed as SI = IC_50_ of extracts on macrophages/IC_50_ of the same extract on promastigotes.

### 2.15. Hemolytic Assay

Blood from healthy subjects was collected in a heparinized vacuum tube, and the erythrocytes were washed 3 times with PBS (centrifugation at 1000 rpm for 10 minutes). A 2% red cell suspension was prepared with PBS. Sample concentrations of 0.5, 0.25, 0.1, and 0.025 *μ*g/mL were incubated with 2% red cells in PBS in the proportion of 1 : 1 in 96-well plates for 3 hours at 37°C/5% CO_2_. PBS was used as a negative control, and distilled water was used to control hemolysis. The plates were centrifuged at 1000 rpm for 10 minutes, and supernatants were collected and analyzed for absorbance reading at 550 nm. This experiment was performed in duplicate and repeated three times.

### 2.16. Statistical Analysis

All of the experiments were performed in triplicate, and the results were expressed as standard error of the mean (SEM) to leishmanicidal analysis and as the mean ± standard deviation (SD) to other analyses. Results were contrasted with a negative and a positive control. The differences between the values were examined using analysis of variance (ANOVA) followed by Tukey's test for multiple comparisons and a *p* value < 0.05 was considered to be statistically significant. Data were analyzed in GraphPad Prism 6.01 software for Windows (GraphPad Software, San Diego, California, USA).

## 3. Results and Discussion

In the recent decades, development of synthetic drugs caused disaffection towards natural products as an attractive resource for searching for new chemotherapy compounds. However, the emergence of some limitations in the use of synthetic drugs as high toxicity, side effects, and elevated costs caused a shift in the situation and interest in the field of ethnobotanical research [20, 25, 26].

Plant-derived natural products are valuable sources in traditional medicine because they have fewer side effects, low cost, and high availability [27]. Indeed, numerous plant-derived bioactive compounds that display a wide variety of pharmacological effects include quercetin and its glucosides rutin and isoquercitrin, common flavonoids, which display antifungal and antileishmanial activities [16, 28, 29].

The* C. coriaceum* extracts stand out due to the high antioxidant action of the pulp, which contains carotenoids and phenolic compounds [30]. In phytochemical qualitative screening, the extracts of* C. coriaceum* showed similar constituents for both extracts presenting the secondary metabolites alkaloids, steroids, saponins, tannins, phenols, and flavonoids, in part corroborating with Araruna et al. [31]. Catechins were present only in pulp extract. In phytochemical tests, both extracts showed positive results to flavonoids, which were analyzed by HPLC, and quercetin, rutin, and isoquercitrin were found as main flavonoids present in the extracts.

The fruit peel extract displays a higher amount of phenols, with 55.617 ± 7.92 mg of gallic acid equivalent (GAE)/g of extract (mg GAE/g) and flavonoids with 3.881 ± 0.10 mg quercetin equivalent (QE)/g of extract (mg QE/g) when compared with fruit pulp extract which showed 24.539 ± 3.55 GAE/g plus 1.334 ± 0.21 QE/g, as observed in Table 1.

Quantification of total phenols and flavonoids by spectrophotometry and HPLC analysis for identification of the main flavonoids from these extracts is shown in Table 1. Isoquercitrin bioavailability was shown to be higher than the other two flavonoids. Rutin is present in a lower amount in both extracts and quercetin was present only in the pulp extract.

The presence of active flavonoids in both extracts explains the biological activities found in this study, which was based on* in vitro* and* in silico* predictions with natural products [32]. Antioxidants could have a pathogen neutralizing action, directly by scavenging ROS or indirectly by activating pathways that promote ROS degradation [27].

Table 2 displays the results of the extracts and respective standard substances' biological activities, as antioxidant, AChE inhibition, and antifungal. The potential of the pulp extract and peel extracts to inhibit free radicals was evaluated. Radical inhibition was higher with increasing concentration of the extracts when compared to the standard (rutin). Comparing* C. coriaceum* fruit part extracts, the pulp presented significantly higher antioxidant potential. Regarding the capacity of scavenging free radicals of the extracts, pulp and peel of* C. coriaceum* fruit showed a better action than that obtained in other plant extracts reported by Moura et al. [33], Penido et al. [20], and Morais et al. [10].

Other studies connected antioxidant mechanisms of action with* in vitro* fungicidal activities of natural compounds [14]. By Holetz's antimicrobial activity index [25], all the extracts can be classified as good antifungals. The ethanol extracts of* C. coriaceum* fruit were effective against six animal pathogenic strains: three of the genus* Malassezia* sp. and three strains of* M. canis*. The MIC and MFC varied between 39.1 and 4.1 in tested microorganisms' strains. However, all the extracts have better results, when compared to other extracts or isolated compounds elsewhere [7, 8].

Against* M. canis*, the tested extracts obtained better results. Peel extract exhibits greater results than pulp extract that was also considered with high activity. Against* Malassezia* spp., both extracts exhibit the same MFC and peel extract demonstrates better MIC than pulp extract (Table 2).

With regard to antiacetylcholinesterase activities, the inhibition zone of pulp was greater than peel extract and similar to the control, physostigmine, which means good AChE inhibition. Other studies [16] determined that the isolated flavonoids found and quantified in this study have a remarkable antiacetylcholinesterase activity and indicate this assay as appropriate to antileishmanial studies. This result indicates that probably there is a direct relationship between inhibition of acetylcholinesterase and antileishmanial action.

Regarding leishmaniasis, the interaction between parasites and host immune cells leads to an inflammatory response essential for parasite control. However, an exacerbated proinflammatory response may cause tissue damage, resulting in lesion formation observed in cutaneous leishmaniasis [34, 35]. On the other hand, the lack of an effective inflammatory response may promote increased parasite burden [26]. In this scenario, the antioxidant effect of* C. coriaceum* extracts can control the inflammatory response being ideal for an effective control of the disease.

Table 3 displays the inhibitory concentration of* Caryocar coriaceum* fruit extracts on promastigote forms of* L. amazonensis* (IC_50_) after 3 days of treatment, toxicity to peritoneal macrophages (CC_50_), and selectivity index (SI) after 24 h of treatment. IC_50_ in parasite proliferation was shown to be similar after 24 h of treatment for both extracts and the standards. At 48 h, there was yet no statistical difference between the treatments. A dose-dependent reaction was found after 72 h with IC_50_ results reduced.

To test the selectivity of pulp and peel extracts, murine macrophages were treated with different concentrations of extracts and the viability of these cells was assessed by the MTT reduction. Pulp extract induced 50 percent of cytotoxicological effect (CC_50_) in peritoneal macrophages at lower concentration than peel at 24 h, but both extracts presented statistically the lowest toxicity compared to the standard (pentamidine). In addition, pulp extract presented a good predilection of the extract by the parasites, being better selective than pentamidine. Then, the* C. coriaceum* extracts had higher toxicity to* L. amazonensis* promastigote forms and demonstrated lower cytotoxicity on murine macrophages and erythrocytes and statistically better results than demonstrated in previous studies for pentamidine and glucantime as standards [9, 17].

Another way to evaluate the cytotoxicity of extracts is by the ability to cause hemolysis in human erythrocytes. As shown in Table 4, the pulp and peel extracts showed low toxicity. The lowest concentration determines hemolytic activities at 0.909 ± 0.746 and 0.616 ± 0.224 per cent, respectively, though with very low levels of hemolysis. The* C. coriaceum* fruit extracts presented low hemolytic activity, and concentrations of 0.1, 0.05, and 0.025 *μ*g/mL were not able to cause significant hemolysis.

## 4. Conclusions

The secondary metabolites produced by* C. coriaceum* are potentially bioactive substances acting as antifungal and antileishmanial agents, mainly by scavenging free radicals and anticholinesterase mechanisms. The flavonoids quercetin, rutin, and isoquercitrin are important indicators of these activities. The results obtained in this study corroborate the potential of these plant species and may form the basis for new antifungal and antileishmanial agents. Nevertheless, further studies are necessary for the isolation and characterization of other substances and* in vivo* studies should be performed to detect the bioavailability of these extracts, aiming at the development of possible new pharmaceutical products.

## Figures and Tables

**Table 1:** Quantification of total phenols and flavonoids by spectrophotometry and main flavonoids by high-performance liquid chromatography (HPLC) of *Caryocar coriaceum *fruit ethanol extracts.

Samples	Total phenols (mg GAE/g)	Total flavonoids (mg QE/g)	Quercetin	Rutin	Isoquercetin
Pulp extract	24.539^b^ ± 3.55	1.334 ± 0.21^b^	1.965^a^	5.025^a^	47.665^b^
Peel extract	55.617^a^ ± 7.92	3.881 ± 0.10^a^	—	4.169^b^	129.198^a^

Similar small letters indicate significant similarities between rows (*p* < 0.0001, according to ANOVA followed by Tukey's test). —: no result.

**Table 2:** Biological activities of *C. coriaceum *extracts.

Extracts	Antioxidant activity (IC_50_ *µ*g/mL)	AChE inhibition (mm)	Antifungal assay *Malassezia* spp. MFC/MIC	Antifungal assay *Microsporum canis* MFC/MIC
Pulp	49.4 ± 0.29^c^	9.0^a^	39.06 ± 1.7^c^/19.53 ± 1.08^b^	9.77 ± 0.12^b^/4.88 ± 0.09^a^
Peel	25.5 ± 0.26^b^	8.5^b^	39.06 ± 0.18^c^/9.77 ± 0.03^a^	4.88 ± 0.06^a^/4.88 ± 0.03^a^
Rutin	13.7 ± 0.25^a^	—	—	—
Physostigmine	—	9.0^a^	—	—

Similar letters indicate significant similarities between rows (*p* < 0.05, according to ANOVA followed by Tukey's test). For MFC/MIC assays, similar letters indicate significant similarities between rows (*p* < 0.0001, according to ANOVA followed by Tukey's test). Rutin: positive control to antioxidant activity. Physostigmine: positive control to AChE inhibition. —: not performed.

**Table 3:** Inhibitory concentration of *Caryocar coriaceum *fruit extracts on promastigote forms of *L. amazonensis *(IC_50_) after 24, 48, and 72 h of treatment, toxicity to peritoneal macrophages (CC_50_), and selectivity index (SI) after 24 h of treatment. Values are expressed in *μ*g/mL.

	24 h	CC_50_	SI	48 h	72 h
	IC_50_			IC_50_	IC_50_
Pulp extract	30 ± 5.0^a^	253 ± 42.0^b^	8.43^b^	39 ± 8.0	17 ± 7.0
Peel extract	38 ± 13.0^a^	454 ± 11.0^a^	11.94^a^	31 ± 9.0	22 ± 3.0
Pentamidine [9]	23.71^a^ (18.44–30.50)	17.90^c^ (0.02–0.03)	0.75^c^		
Glucantime [17]	13.95 (±2.06)^a^				

Data represent the mean ± SEM of at least three independent experiments performed in triplicate. CC_50_: cytotoxic concentration of 50% of macrophages (*µ*g/mL). IC_50_: inhibitory concentration of 50% of promastigote forms (*µ*g/mL). SI: selectivity index = CC_50_/IC_50_. Similar letters indicate significant similarities between rows (*p* ≤ 0.05, according to ANOVA followed by Tukey's test).

**Table 4:** Hemolytic activity (%) of *Caryocar coriaceum *fruit extracts (mg/mL).

	0.025	0.05	0.1	0.25	0.5
Pulp extract	0.909 (±0.746)	1.300 (±0.341)	1.295 (±0.564)	2.406 (±1.206)	8.777 (±4.102)
Peel extract	0.616 (±0.224)	1.229 (±0.740)	0.925 (±0.315)	3.068 (±1.368)	6.872 (±3.056)

Data represent the mean ± SEM of at least three independent experiments performed in triplicate. Data were normalized to the positive control (distilled water).
